# TGFβ-induced osteogenic potential of human amniotic fluid stem cells via CD73-generated adenosine production

**DOI:** 10.1038/s41598-017-06780-1

**Published:** 2017-07-26

**Authors:** Kwan-Leong Hau, Anna Maria Ranzoni, Filipa Vlahova, Kate Hawkins, Paolo De Coppi, Anna L. David, Pascale V. Guillot

**Affiliations:** 1 National Heart & Lung Institute, Hammersmith Campus, Du Cane Road, Imperial College London, London, W12 0NN UK; 2 Institute for Women’s Health, Research Department of Maternal and Fetal Medicine, University College London, 86-96 Chenies Mews, London, WC1E 6HX UK; 3 Great Ormond Street Institute of Child Health, University College London, 30 Guilford Street, London, WC1N 1EH UK

## Abstract

The human amniotic fluid stem cell (hAFSC) population consists of two morphologically distinct subtypes, spindle-shaped and round-shaped cells (SS-hAFSCs and RS-hAFSCs). Whilst SS-hAFSCs are routinely expanded in mesenchymal-type (MT) conditions, we previously showed that they acquire broader differentiation potential when cultured under embryonic-type (ET) conditions. However, the effects of culture conditions on RS-hAFSCs have not been determined. Here, we show that culturing RS-hAFSCs under ET conditions confers faster proliferation and enhances the efficiency of osteogenic differentiation of the cells. We show that this occurs via TGFβ-induced activation of CD73 and the associated increase in the generation of extracellular adenosine. Our data demonstrate that culture conditions are decisive for the expansion of hAFSCs and that TGFβ present in ET conditions causes the phenotype of RS-hAFSCs to revert to an earlier state of stemness. Cultivating RS-hAFSCs in ET conditions with TGFβ may therefore increase their therapeutic potential for clinical applications.

## Introduction

Human amniotic stem cells (hAFSCs) hold great therapeutic potential in regenerative medicine because they can be isolated from mid-trimester and term amniotic fluid samples without ethical restrictions, and are developmentally more primitive than adult stem cells^1–3^.

The hAFSC population comprises two subpopulations of plastic-adherent multipotent cells that are morphologically distinct, i.e. fast-growing spindle-shaped cells (SS-hAFSCs) and slow-growing round-shaped cells (RS-hAFSCs)^4, 5^. Populations of plastic-adherent multipotent spindle-shaped and round-shaped (also referred to as flat-shaped) stem cells have already been described in umbilical cord tissue (UC)^6^. Like SS-hAFSCs, UC-derived spindle-shaped stem cells only constitute a minority of cells, whilst round-shaped cells are present in greater proportion^6^.

SS-hAFSCs are routinely isolated and expanded under mesenchymal-type (MT) conditions, i.e. either in Dulbecco’s Modified Eagle’s Medium (DMEM), α-minimum essential medium (α-MEM) or Chang medium B and C supplemented with 5–15% fetal bovine serum (FBS), designed to sustain the phenotype of adult mesenchymal stem cells (MSCs)^3, 5, 7^. However, hAFSCs have been described as having a privileged intermediate phenotype between embryonic stem cells (ESCs) and adult MSCs. This is because they express the standard MSC markers and can be expanded to large numbers without the need for feeder cell layers^8, 9^, but they also exhibit broader differentiation potential, longer telomeres and more active telomerase activity than adult MSCs^10, 11^.

We previously showed that switching the culture conditions of SS-hAFSCs from MT conditions to conditions used to maintain the pluripotent state of ESCs (ET) induced important phenotypic phenotype changes such as greater proliferation kinetics, reactivation of some markers associated with pluripotency and the ability to form embryonic bodies^10, 12^. However, RS-hAFSCs, which are routinely expanded in MT conditions, have been described as slow-growing cells with limited differentiation potential compared to their SS-hAFSC counterparts ^4, 5^.

Here, we investigated whether culturing RS-hAFSCs in ET culture conditions, either from the time of isolation from the amniotic fluid samples or following initial expansion in MT culture conditions, would positively impact on the phenotype of the cells. We show that ET culture conditions stimulate the expansion kinetics of RS-hAFSCs and promote the efficiency of osteogenic differentiation of the cells via transforming growth factor (TGF)β-induced activation of CD73 and the associated increase in adenosine production.

## Results

### ET culture conditions reduced the cell size and increased growth kinetics and alkaline phosphatase expression in RS-hAFSCs

**Figure 1:**
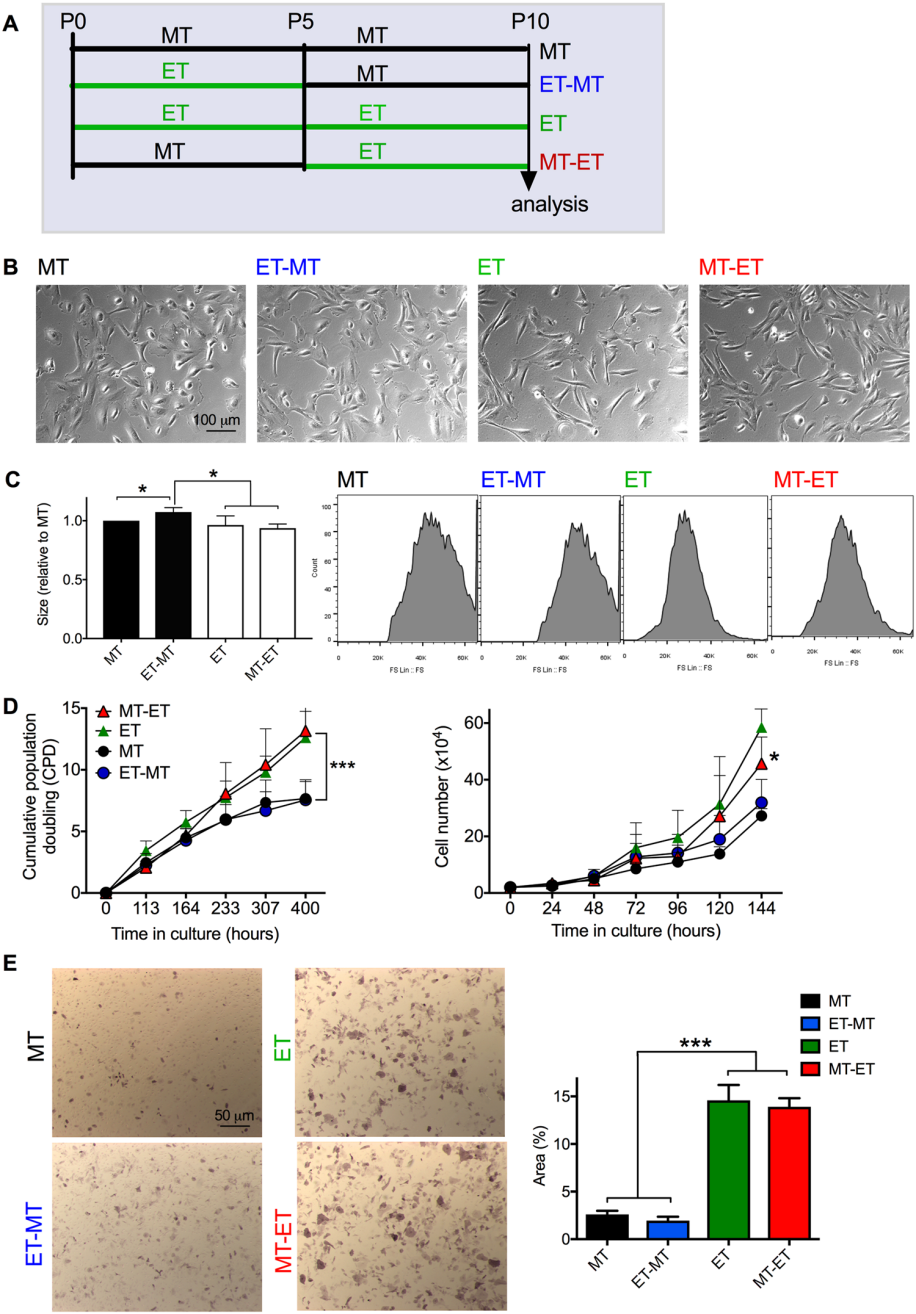
Characterization of human round-shaped amniotic fluid stem cells (RS-hAFSCs) cultivated in MT and ET culture conditions. (**A**) Experimental design showing the different culture conditions of RS-hAFSCs, split into four experimental groups. Two groups of cells were cultured from the time of isolation until passage 10, one group in mesenchyme type (MT) culture conditions (on uncoated plastic dishes in DMEM supplemented with 10% FBS) and the other in embryonic type (ET) culture conditions (on Matrigel-coated plastic dishes in Nutristem media). The remaining two groups were cultured in either MT or ET conditions until passage 5, when the culture conditions were switched to ET conditions in one group (MT-ET) and MT conditions in the other group (ET-MT). The cells were analysed at passage 10. (**B**) Phase contrast images of RS-hAFSCs showing the morphology of the cells maintained either in MT, ET, MT-ET or ET-MT conditions. (**C**) Relative size of RS-hAFSCs cultured in MT, MT-ET, ET or ET-MT culture conditions (mean value of 3 median values each calculated from 10,000 cells). Representative flow scatter showing differences in forward scatter (FSC) median. Data represent mean ± SEM. SEM: standard error of the mean. *P < 0.05. (**D**) Left hand graph: cumulative population doubling calculated over 20 days for 3 different hAFSC samples cultured in different conditions. Right hand graph: cumulative cell numbers were calculated over 144 hours in the four different experimental groups. Data represent mean ± SEM. ***P < 0.001. (**E**) Alkaline phosphatase activity showing % area of positive stain and representative images for RS-hAFSCs cultured in different culture conditions. Data represent mean ± SEM. ***P < 0.001.

To assess whether the culture conditions of RS-hAFSCs affects their phenotype and determine the optimal culture conditions for *in vitro* expansion, we isolated and subsequently maintained RS-hAFSCs either in mesenchyme type (MT) conditions or human embryonic stem cells (ET) conditions on Matrigel-coated plates in Nutristem media. We also isolated and expanded RS-hAFSCs in MT or ET culture conditions until the cells reached passage 5, at which time the cells expanded in MT conditions were switched to ET conditions (MT-ET) and *vice versa* (ET-MT) for another 5 passages (Fig. 1A). Three different hAFSC samples were analysed throughout the study. RS-hAFSCs exhibited a round-shaped morphology under each culture conditions (Fig. 1B), although the size of cells cultivated in ET conditions (ET and MT-ET conditions) was smaller than the size of the cells cultivated in MT conditions (Fig. 1C).

RS-hAFSCs expanded wholly or subsequently in ET conditions (ET or MT-ET) showed higher growth kinetics than cells expanded in wholly or subsequently in MT conditions (MT or ET-MT), as evidenced by higher cumulative population doubling (CPD) (7.6 ± 0.8 and 7.5 ± 0.8 vs. 12.6 ± 1.2 and 13.2 ± 1.3, p < 0.001) over the 16 days of analysis (Fig. 1D). These results indicate that growing RS-hAFSCs in ET conditions favors their expansion and survival.

In non-pluripotent stem cells, alkaline phosphatase (AP) is associated with proliferation^13^, and is a mark of osteogenic progenitors^14, 15^. We found that AP was not expressed in RS-hAFSCs cultured wholly or subsequently in MT conditions (MT or ET-MT), but culturing the cells wholly or subsequently in ET conditions (ET or MT-ET) induced AP expression, even when the cells have been isolated and initially expanded in MT conditions (Fig. 1E).

### ET culture conditions increased CD73 expression in RS-hAFSCs

**Figure 2:**
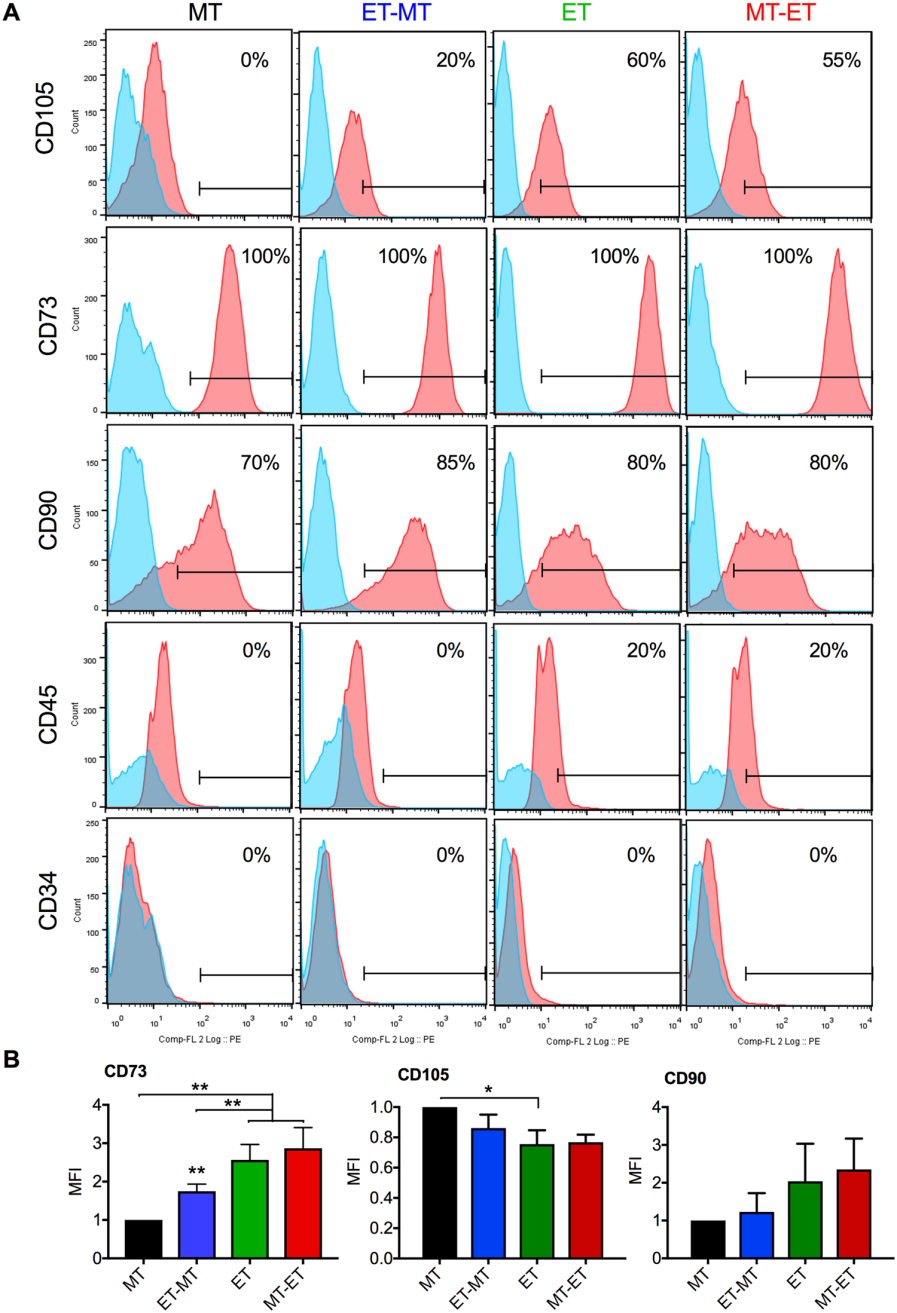
Immunophenotype of RS-hAFSCs cultured under different conditions. (**A**) Flow cytometry to show levels of expression of CD105, CD73, CD90, CD45 and CD34. Isotype control in blue. (**B**) Relative mean fluorescence intensity (MFI) for the different groups (measured from 10,000 cells and using the same antibody concentration, experimental conditions and cytometer settings for all groups). Data represent mean ± SEM. SEM: standard error of the mean. **P < 0.01.

CD105 (endoglin), a type I membrane glycoprotein that plays an important role in the modulation of the TGFβ response, cellular migration and inflammation^16^, was not expressed in RS-hAFSCs cultured wholly in MT conditions. Conversely, cells expanded in ET conditions showed intermediate phenotype with highest proportions of positive cells when cultured wholly in ET conditions (Fig. 2A). CD73 (ecto-5′-nucleotidase), which plays a crucial role in the production of adenosine, was homogeneously expressed in cells for the four culture conditions tested (Fig. 2A). However, comparative analysis of the mean fluorescence intensity (MFI) showed CD73 was more highly expressed on cells cultured wholly or subsequently in ET conditions (ET and MT-ET) in comparison to cells cultures wholly or subsequently in MT conditions (MT and ET-MT) (Fig. 2B). CD90 (Thy-1), which plays a role in T-cell activation, cell adhesion and migration, was expressed at similar levels under all conditions tested (Fig. 2A). The cells did not express CD45, a tyrosine phosphatase also known as the leukocyte common antigen (LCA) or the hematopoietic progenitor cell antigen CD34 in either conditions (Fig. 2A).

**Figure 3:**
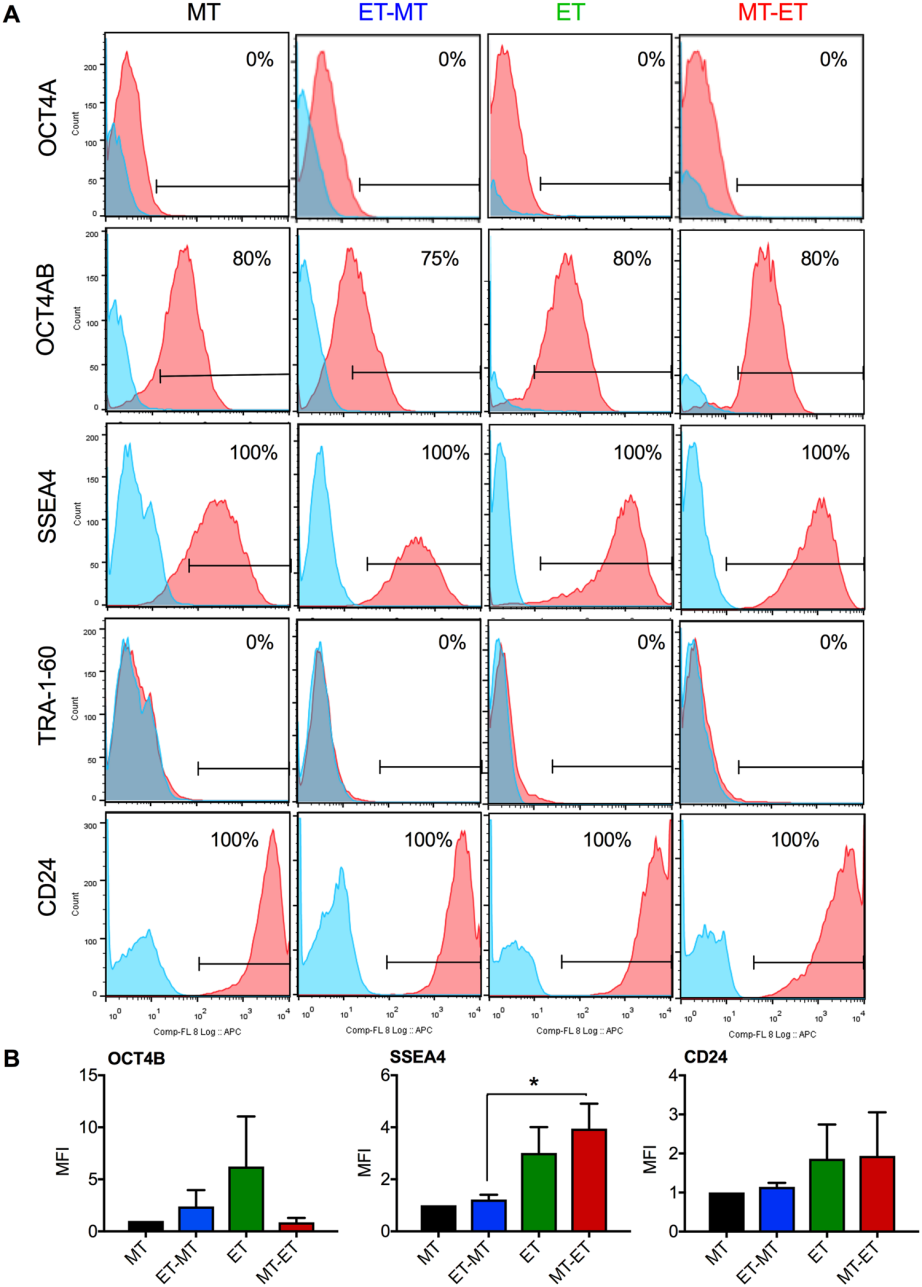
Immunophenotype of RS-hAFSCs cultured in in different conditions. (**A**) Flow cytometry to show the expression of OCT4-A, OCT4-B, SSEA4, Tra1-60, and CD24. Isotype control in blue. (**B**) Relative mean fluorescence intensity (MFI) for the different groups (measured from 10,000 cells and using the same antibody concentration, experimental conditions and cytometer settings for all groups). Data represent mean ± SEM. SEM: standard error of the mean. **P < 0.01.

The human *OCT4* (octamer-binding transcription factor 4) gene also known as POU5F1, generates 3 transcripts (OCT4A, OCT4B and OCT4B1) by alternative splicing^17^. OCT4A, a key transcription factor involved in maintaining the pluripotency and self-renewal of ESCs and induced pluripotent stem cells (iPSCs), was not expressed in RS-hAFSCs, regardless of their culture conditions; although OCT4B, which does not sustain pluripotency and is expressed at low level in human somatic stem cells, tumour cells and adult tissues^17^, was expressed with a similar intensity in all conditions (Fig. 3A and B). SSEA4 (stage-specific embryonic antigen-4), a cell surface marker commonly used to identify pluripotent cells also expressed in the adult MSC population^18^, was expressed in all groups (Fig. 3A), with cells cultured wholly or subsequently in ET or MT-ET conditions showing higher expression (Fig. 3B). TRA-1-60 (T cell receptor alpha), a stem cell marker associated with pluripotency in human ESCs and iPSCs that is lost with differentiation, was absent in all groups (Fig. 3A). Signal transducer CD24 (cluster of differentiation 24) is a cell surface adhesion molecule expressed in ESCs, neural stem cells and MSCs, where it modulates TGFβ signaling^19^. CD24 was expressed in all groups with a similar intensity (Fig. 3A and B).

### ET culture conditions increased *in vitro* osteogenic differentiation efficiency of RS-hAFSCs

**Figure 4:**
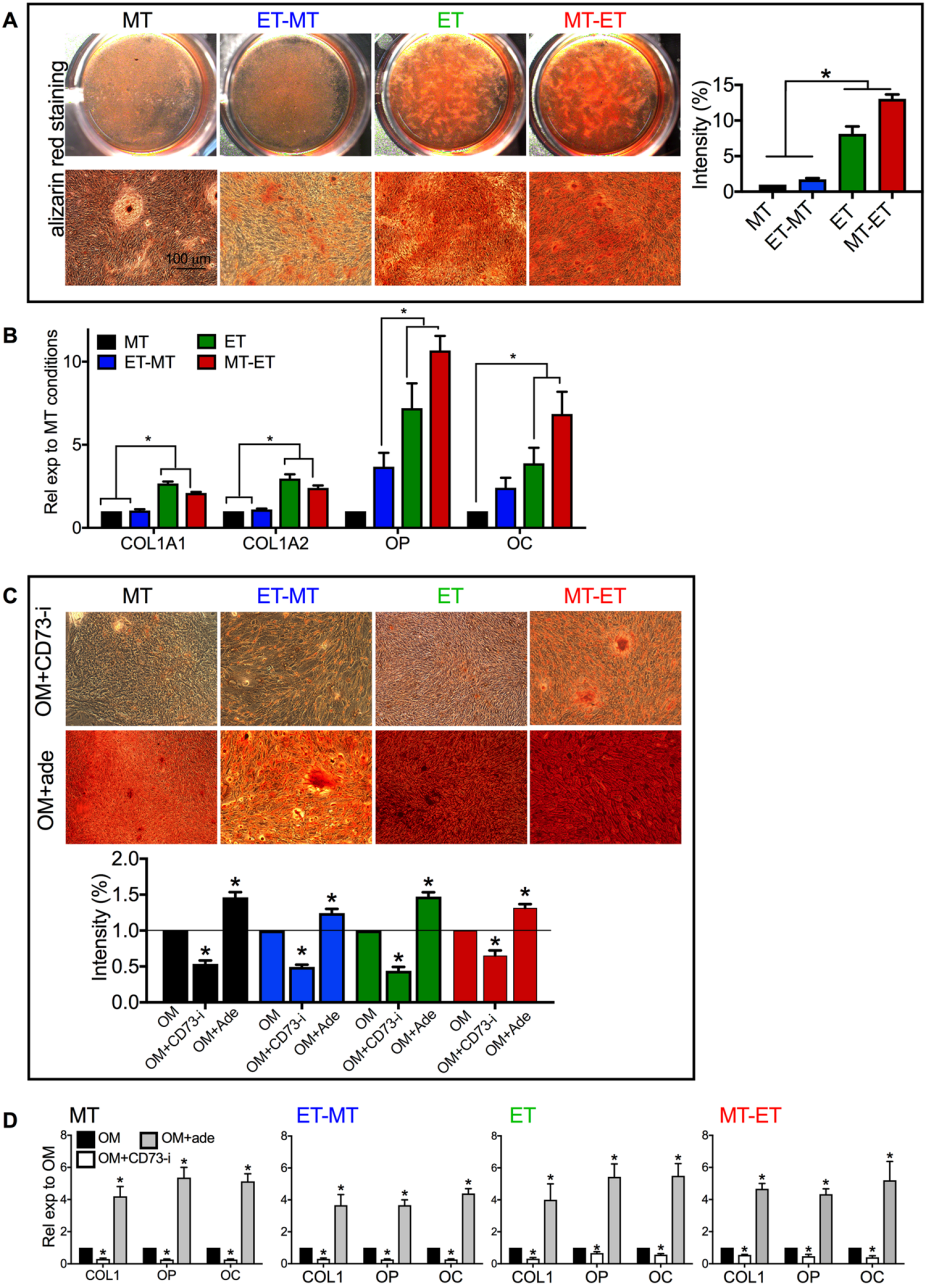
*In vitro* osteogenic differentiation of RS-hAFSCs. (**A**) The media of passage 10 RS-hAFSCs cultured in different conditions was replaced by osteogenic medium (OM) for 3 weeks. The cultures were subsequently stained with Alizarin red to visualize areas of calcium deposition. The graph on the right hand represents the staining intensity of alizarin red staining relative to MT condition. (**B**) qRT-PCR to show expression of the osteogenic genes COL1A1, COL1A2, OP and OC in the four groups. GAPDH was used as internal control. (**C**) Passage 10 cells were cultured in osteogenic media supplemented with either the CD73 inhibitor adenosine 5′-(α,β-methylene) diphosphate (OM+CD73i) or with the CD73 downstream product adenosine (OM+ade) for 3 weeks and subsequently stained with Alizarin red. The bottom panel represents the staining intensity relative to all condition. (**D**) qRT-PCR for collagen type I (*COLI*), osteocalcin (*OC*) and osteopontin (*OP*), normalized by their expression in RS-hAFSCs cultured in MT conditions. GAPDH was used as internal control. Data represent mean ± SEM for 3 samples per group. SEM: standard error of the mean. *P < 0.001. (mean ± SEM).

CD73-generated adenosine has been shown to promote osteoblast differentiation^20–22^ and SSEA4+ stem cell sub-populations exhibit enhanced osteogenic differentiation potential^23^. Therefore, we investigated the effect of culture conditions on osteogenic differentiation potential. RS-hAFSCs were cultured in ET or MT conditions for 10 passages, detached and reseeded as single cells onto uncoated plastic dishes in osteogenic medium for 3 weeks. Cells that were wholly or subsequently cultured in ET conditions (ET or MT-ET) produced greater amounts of calcium (Fig. 4A) and showed higher levels of expression for the osteogenic genes COL1A, COL1A2, OC and OP (Fig. 4B). These results indicated that ET culture conditions are associated with higher levels of CD73 and a 4.4 time increase in osteogenic differentiation efficiency.

### ET culture condition enhanced osteogenic differentiation efficiency of RS-hAFSCs via CD73 expression

To test whether CD73 directly modulated osteogenic differentiation potency of RS-hAFSCs, the osteogenic medium was supplemented daily with the CD73 inhibitor adenosine 5′-(α,β-methylene) diphosphate (CD73-i). As extracellular adenosine is generated by the dephosphorylation of adenosine triphosphate (ATP) by CD73, we also tested whether supplementation of the osteogenic medium with adenosine would enhance the osteogenic differentiation efficiency of RS-hAFSCs.

In all four groups, calcium deposition, visualized by alizarin red staining, was abrogated when the cells were treated with CD73 inhibitor, but increased when the medium was supplemented with adenosine (Fig. 4C). Analysis of the expression of the osteogenic genes collagen type I (*COL1*), osteopontin (*OP*) and osteocalcin (*OC*) confirmed that inhibition of CD73 prevented osteogenic differentiation of RS-hAFSCs, whilst adenosine supplementation increased the expression of the late osteogenic markers COL1, OP and OC (Fig. 4D). Taken together, these results indicate that ET culture conditions increase the osteogenic differentiation efficiency of RS-hAFSCs by 150% by stimulating CD73-induced adenosine production.

### TGFβ induced nuclear import of Smad2/3 and increased CD73 expression in RS-hAFSCs cultured in MT conditions

**Figure 5:**
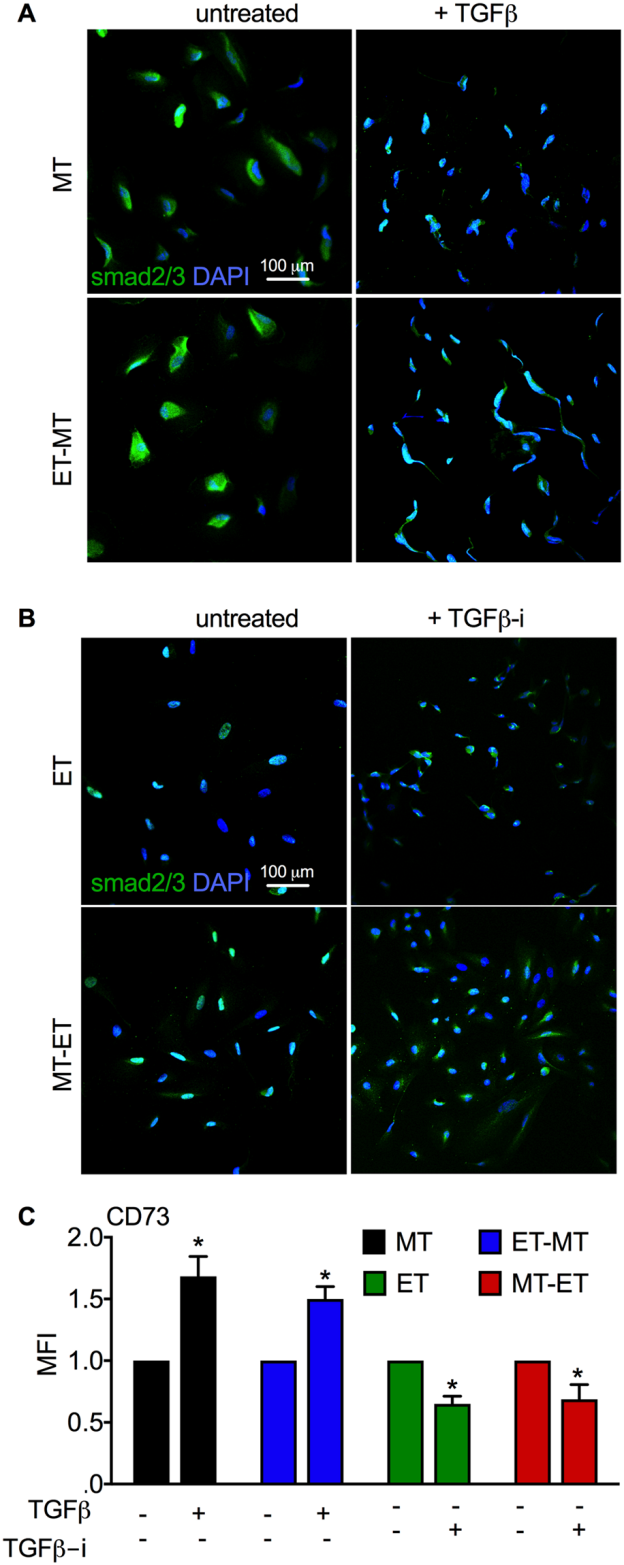
TGFβ signaling in RS-hAFSCs cultured in different conditions. Confocal immunofluorescence staining for SMAD2/3. Nuclei were stained with DAPI (blue). (**A**) RS-hAFSC cells were cultured in MT or ET-MT with and without (untreated) supplementation with TGFβ. (**B**) RS-hAFSC cells were cultured in ET or MT-ET with and without (untreated) supplementation with a TGFβ inhibitor (TGFβ-i). (**C**) Mean fluorescence intensity (MFI) for the different groups (measured from 10,000 cells and using the same CD73 antibody concentration, experimental conditions and cytometer settings for all groups). Data represent mean ± SEM. SEM: standard error of the mean. **P < 0.01.

CD73 expression and adenosine generation are up-regulated by the TGFβ-Smad2/3 signaling pathway^24^ and the translocation of Smad2/3 from the cytoplasm to the nucleus is an indication of the activation of TGF-β signaling. Smad2/3 is localised in the cytoplasm of RS-hAFSCs cultured wholly or subsequently in MT conditions (MT and ET-MT) which express low levels of CD73 (Fig. 5A), but in the nucleus of cells cultured wholly or subsequently in ET conditions (ET and MT-ET) expressing high levels of CD73 (Fig. 5B). This suggests that CD73 is modulated by TGFβ in RS-hAFSC and that ET culture conditions may increase the osteogenic potency of the cells by stimulating CD73 expression. To test this hypothesis, we treated the cells cultured in MT conditions with TGFβ and the cells cultured wholly or subsequently in ET conditions with a TGFβ inhibitor. We found that TGFβ treatment induced nuclear import of Smad2/3 (Fig. 5A) whilst TGFβ inhibitor treatment induced its nuclear export (Fig. 5B). We next confirmed that TGFβ-induced Smad2/3 nuclear import was associated with an increase in CD73 expression, whilst TGFβ inhibitor-induced Smad2/3 nuclear export was associated with a decrease in CD73 expression (Fig. 5C)

### TGFβ treatment enhanced osteogenic differentiation efficiency of RS-hAFSC

**Figure 6:**
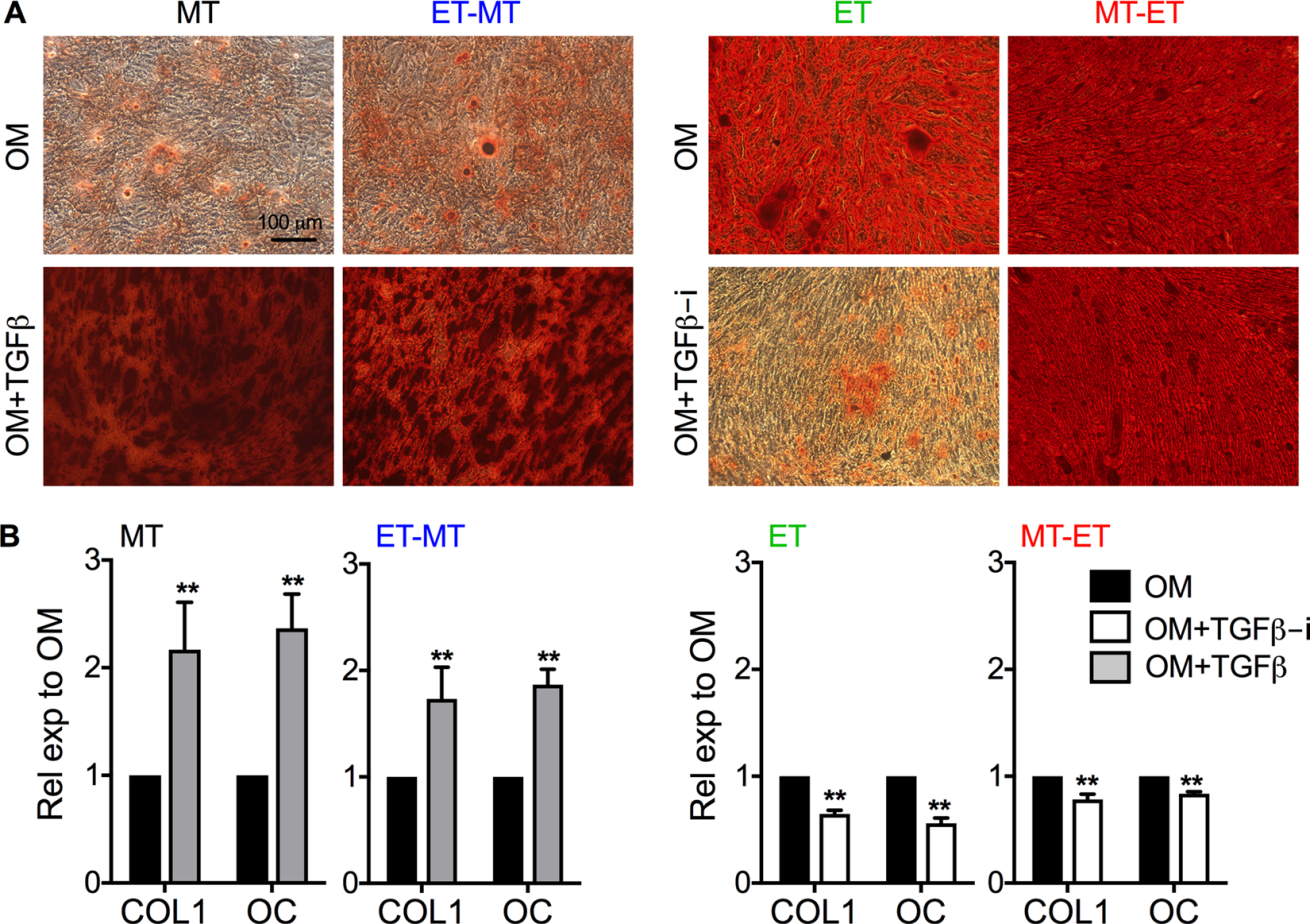
TGFβ-dependent modulation of RS-hAFSC osteogenic differentiation. (**A**) The media of passage 16 RS-hAFSCs cultured in different conditions was replaced by osteogenic medium (OM) for 3 weeks. Alternatively, the media of passage 10 RS-hAFSCs cultured in MT or ET-MT conditions was supplemented with TGFβ for 10 days before being replaced by osteogenic medium (OM+TGFβ) for 3 weeks and the media of passage 10 RS-hAFSCs cultured in ET or MT-ET conditions was supplemented with a TGFβ inhibitor (OM+TGFβ-i) for 48 h before being replaced by osteogenic medium (OM) for 3 weeks. The cultures were subsequently stained with Alizarin red to visualize areas of calcium deposition. (**B**) qRT-PCR for the osteogenic genes collagen type I (*COL*) and osteoclacin (*OC*), normalized to their expression in RS-hAFSCs cultured under MT conditions. GAPDH was used as internal control.

Since cells expressing high levels of CD73 showed higher osteogenic differentiation potency and TGFβ treatment increased CD73 levels, we next investigated whether treatment of RS-hAFSCs with TGFβ would lead to an increase in osteogenic differentiation potential. We observed that treatment with TGFβ of RS-hAFSC after culture in MT conditions increased calcium deposition (Fig. 6A) and expression levels of the osteogenic genes COL1 and OC (Fig. 6B), whilst treatment with a TGFβ inhibitor of the cells cultured in ET conditions prevented osteogenic differentiation (Fig. 6A and B). Together, these data indicate that ET culture conditions increase the osteogenic differentiation efficiency of RS-hAFSCs via TGFβ-induced increase in CD73-mediated adenosine production.

## Methods

### Ethics statement

The healthy donors who provided mid-trimester amniotic fluid in this study gave written informed consent in accordance with the Declaration of Helsinski. The ethical approval given by the joint Research Ethics Committee of University College London (UCL) and UCL Hospital (Ethics of Human Research) approved amniotic fluid stem cell collection (REC 08/H0714/87), in compliance with UK national guidelines (Review of the Guidance on the Research Use of Fetuses and Fetal Material (1989), also known as the Polkinghorne Guidelines. London: Her Majesty’s Sationery Office, 1989:Cm762) for the collection of human fetal tissue for research.

### Cell culture

Amniocentesis procedures were performed under sterile conditions for clinical reasons such as prenatal diagnosis, and surplus fluid was used for research purposes. All amniotic fluid samples had a normal karyotype. Human amniocytes (hAFSCs) were isolated from the amniotic fluid (AF) between 15 to 22 weeks of gestation (healthy pregnancy). Fresh samples were divided in two. The AF was then pelleted and resuspended as a single cell suspension either on uncoated plastic dishes in Dulbecco’s Modified Eagle’s Medium (DMEM-HG) (Invitrogen) supplemented with 10% fetal bovine serum (FBS) (Biosera), 2 mM L-Glutamine, 50 IU/ml penicillin and 50 mg/ml streptomycin (Gibco-BRL) (defined as MT culture conditions); or on Matrigel (BD) coated dishes in ESC media, i.e. Nutristem XF/FF (Stemgent) (defined as ET culture conditions). After 7–14 days of culture, single colonies appeared, with clones of cells presenting either a round-shape or a spindle-shape morphology. Non-adherent cells were removed by washing the cell culture with PBS. The colonies with ≥50 cells were trypsinised and collected as described by Roubelakis *et al*.^4^ (Supplementary Figure 1). The colonies of round-shape (RS) cells were pooled and resuspended in either MT or ET conditions. Alternatively, the cells were cultured in either MT or ET conditions over the first 5 passages, and were subsequently transferred to ET or MT conditions, respectively (MT-ET and ET-MT conditions). The cells were cultured for a further 5 passages. All cells were analysed at passage 10. The cells were maintained at 37 °C in a 5% CO_2_ incubator. Alkaline phosphatase staining was performed following the manufacturer’s protocol (Roche).

### Growth kinetics

The growth rate of RS-hAFSCs was estimated by plating 20,000 cells per well in a 12-well tissue culture plate in triplicate (3 samples). Every 24 hours, the cells were detached and counted in a hemocytometer using trypan blue exclusion of dead cells. To compare the cumulative population doublings of the cells cultured in different conditions, they were plated in triplicate at a concentration of 10^4^ cells per cm^2^ in 10-cm^2^ dishes and successively subcultured at the same density when subconfluent. This replating procedure was serially repeated over 20 days, and the cumulative population doublings were determined by counting the number of adherent cells at the start and end of each passage.

### Flow cytometry

The cells were detached, dissociated into single cells using dispase (Sigma-Aldrich), and resuspended in fluorescence-activated cell sorting (FACS) buffer (phosphate-buffered saline PBS, 1% BSA; 5 × 10^5^ cells/100 μl FACS buffer). For cell-surface staining the cells were stained with primary antibodies CD73-PE (130-095-182, Miltenyi), CD105-FITC (130-094-926, Miltenyi), CD90- PE-VIO770 (130-099-295, Miltenyi), CD45-PE (130-098-141, Miltenyi), CD34-FITC (130-098-139, Miltenyi), CD24-FITC (130-098-861, Miltenyi), SSEA4-PE (FAB1435P, R&D), and TRA1-60-PE (09-0009, Stemgent) for 1 hour at 4 °C. For intracellular FACS staining, i.e. OCT4-A (130-105-606, Miltenyi) and OCT4-B (IC1759P, R&D), the cells were washed once in FACS buffer, fixed for 10 min in 0.01% paraformaldehyde (PFA), washed twice with PBS, resuspended in permeabilization buffer (PBS, 1% Triton) and stained as describe above. Cells were then washed twice with PBS, resuspended in FACS buffer and analysed by FACScalibur flow cytometry (CyAn ADP, Beckman Coulter). Collected data were analyzed with the Flowjo v.10 software package (Treestar, Ashland, OR, USA). Negative control was immunoglobulin G (IgG) primary antibody-specific isotypes.

### Confocal immunostaining

For confocal immunostaining, the cells were directly stained on coverslips placed in the cell culture dishes. The cells were fixed in 4% paraformaldehyde (PFA) (10 min) and permeablized in 0.5% Triton X-100 in PBS (15 min). Cells were then rinsed in PBS, blocked (1 hour) with 5% normal goat serum, incubated (overnight, 4 °C) with primary antibody (smad2/3 1:200, Cell Signaling Technology) in blocking solution, washed in blocking solution, incubated (1 hour) with secondary antibody (goat anti rabbit igG A11008, Thermo Fisher) in blocking solution, rinsed in PBS and stained with DAPI before being mounted in VectaShield (Vector Labs). Images were collected on a confocal laser-scanning microscope (Zeiss LSM7100).

### *In vitro* osteogenic differentiation

Cells were detached, counted and re-seeded in a 24-well plate (with 40,000 cells per well) in osteogenic medium consisting in DMEM supplemented with 10% FBS, 2 mM L-Glutamine, 50 IU/ml penicillin, 50 mg/ml streptomycin, 10^−8^ M dexamethasone, 10^−3^ M ascorbic acid, and 10 mM beta-glycerophophate (all Sigma-Aldrich). Osteogenic medium was freshly prepared and replaced twice weekly for 21 days. To detect mineralized nodules, cell culture plates were fixed in 10% formalin-buffered saline for 1 hour, washed in PBS and stained for 10 min with a 1% solution of alizarin red. Plates were then washed in water and left to air dry. Staining intensity was quantified using Image J software

### Chemical treatment

The CD73 inhibitor adenosine 5′-(α,β-methylene) diphosphate (200 uM, M3763 Sigma-Aldrich), adenosine (10 uM, A9251 Sigma-Aldrich), TGF-β (5 ng/ml, T7039 Sigma Aldrich) and TGF-β inhibitor (1 uM, S4317 Sigma Aldrich) were added freshly to the culture media daily over 3 weeks.

### Quantitative real time RT-PCR

Total RNA was extracted using RNeasy Mini Kit (Qiagen) and cDNA was synthetized using High Capacity cDNA Reverse Transcription Kits (Thermo Scientific), according to the manufacturer’s instructions. The generated cDNA was amplified using the ABI StepOne Sequence Detector system (Applied Biosystems) and SYBR green. Samples were normalized against the internal control (GAPDH).

### Statistical analysis

Comparisons between groups were made using one-way ANOVA followed by Bonferroni’s multiple comparison post hoc test. The *p* values < 0.05 were considered significant. These were calculated using Prism-7(version 7.0b) graphing and statistical software (GraphPad, San Diego, CA, USA). Data were expressed as mean ± SEM (standard error of the mean).

### Data availability

All data generated or analysed during this study are included in this published article (and its Supplementary Information files).

## Discussion

Multipotent hAFSCs represent a heterogeneous population of spindle-shaped and round-shaped stem cells^4, 5^. Despite presenting an intermediate phenotype between adult mesenchymal stem cells (MSCs) and embryonic stem cells (ESCs), SS- and RS-hAFSCs are routinely cultivated in conditions that support the adhesion and expansion of multipotent somatic fetal and adult stem cells (MT culture conditions), i.e. on uncoated plastic dishes either in Chang medium, DMEM or α-MEM supplemented with various concentration of FBS^3^. Whilst hAFSCs proliferate and maintain a stable phenotype in MT culture conditions, little is known about the optimal requirement of these cells to maintain their original *in vivo* phenotype. We previously showed that maintenance of SS-hAFSCs in conditions designed to sustain the pluripotent and self-renewal phenotype of ESCs and induced pluripotent stem cells (iPSCs), supplemented with valproic acid, reverted the cells to an earlier state of stemness, as evidenced by increased growth kinetics, activation of pluripotency genes and broader differentiation potential^10, 12^. The cells did not acquire tumorigenicity under these conditions since they were unable to form teratomas upon subcutaneous infusion into immune-compromised mice^10, 12^. RS-hAFSCs, which are more abundant than their SS counterparts, have been described as slower-growing epithelial-like cells with lesser differentiation potential.

Here, we show that cultivating RS-hAFSCs in ET conditions (either from the time of isolation or after an initial period of expansion in MT conditions) modified the phenotype of the cells and promoted their stemness, as evidenced by reduced cell size, increased growth kinetics and higher expression of alkaline phosphatase, CD73, CD105 and SSEA4. Importantly, this translated into greater *in vitro* osteogenic differentiation efficiency. Analysis of the mechanisms mediating this effect revealed that osteogenic differentiation potency of the cells is promoted through TGFβ-induced activation of CD73-generated extracellular adenosine.

Active TGFβ, which is measured without the acidification step in ELISA, is present in Nutristem (2 ng/ml) but is not detectable in FBS and in DMEM used to culture the cells in MT conditions, and is not produced by the cells (data not shown), and latent TGFβ has no biological activity. The amniotic fluid contains nutrients and growth factors that regulate fetal growth and development, such as TGFβ^25^. Active TGFβ is found in humans during the late stage of gestation and accelerates the rate of healing of intestinal wounds by stimulating cell migration^25^. Therefore, the composition of the Nutristem, which includes TGFβ, may be closer to the composition of the human amniotic fluid and may therefore sustain, or contribute to the cells regaining a more primitive phenotype of the cells, whilst culture in MT conditions may induce the differentiation of the cells and reduce their differentiation and self-renewal potential.

At the cellular level, TGFβ upregulates the cell surface protein expression levels of ecto-5′-nucleotidase (CD73) cell surface levels by activating the NT5E gene via the TGFβ-Smad2/3 pathway^24^. CD73 then dephosphorylates ATP to produce adenosine in the extracellular space^26^. Extracellular adenosine regulates the inflammatory response by suppressing pro-inflammatory cytokines and stimulating anti-inflammatory cytokines^27^. In bone, adenosine released locally regulates the differentiation and function of bone-forming osteoblasts and bone-resorbing osteoclasts^28^. Extracellular accumulation of adenosine results in the activation of adenosine receptors. There are four receptors, i.e. A_1_R, A_2A_R A_2B_R, and A_3_R. Under adenosine conditions, A_1_R is activated to induce osteoclast differentiation, but when adenosine levels are above physiological conditions, the other receptors are activated, which inhibits osteoclast differentiation by decreasing IL1β and TNFα secretion^29^. In contrast, in osteoblasts, activation of A_2A_R increases osteoblast differentiation and bone formation by activating the expression of osteoblast-related genes^28^. Together, these data indicate that the presence of TGFβ in Nutristem promotes the *in vitro* osteogenic differentiation of RS-hAFSCs, providing a strategy to augment the osteogenic potential of the cells for the treatment of bone pathologies.

Our findings also highlight that the phenotype of RS-hAFSCs is reversible, since cells that have been previously expanded in MT culture conditions before being switched to ET conditions acquire similar osteogenic efficiency and growth kinetics as cells that have been isolated and expanded exclusively in ET conditions. These results underscore the ability of hAFSCs to revert to an earlier state of stemness using chemicals alone, in the absence of ectopic expression of foreign DNA or RNA. The plastic phenotype of hAFSCs has already been documented in SS-hAFSCs, which could be reverted to functional pluripotency by cultivating the cells in ET conditions supplemented with the histone deacetylase inhibitor valproic acid^10, 12, 30^. Further studies will be needed to determine the broader effect of ET culture condition on hAFSCs phenotype.

## Electronic supplementary material

Supplementary information

## References

[1] Guillot PV, O’Donoghue K, Kurata H, Fisk NM (2006). Fetal stem cells: betwixt and between. Semin. Reprod. Med..

[2] Guillot PV, Götherström C, Chan J, Kurata H, Fisk NM (2007). Human first-trimester fetal MSC express pluripotency markers and grow faster and have longer telomeres than adult MSC. Stem Cells.

[3] Loukogeorgakis SP, De Coppi P (2016). Stem cells from amniotic fluid–Potential for regenerative medicine. Best Pract Res Clin Obstet Gynaecol.

[4] Roubelakis MG (2011). *In vitro* and *in vivo* properties of distinct populations of amniotic fluid mesenchymal progenitor cells. J. Cell. Mol. Med..

[5] Arnhold S (2011). Amniotic-Fluid Stem Cells: Growth Dynamics and Differentiation Potential after a CD-117-Based Selection Procedure. Stem Cells Int.

[6] Chang Y-J, Tseng C-P, Hsu L-F, Hsieh T-B, Hwang S-M (2006). Characterization of two populations of mesenchymal progenitor cells in umbilical cord blood. Cell Biol. Int..

[7] Moschidou D (2013). Molecular signature of human amniotic fluid stem cells during fetal development. Curr Stem Cell Res Ther.

[8] Dominici M (2006). Minimal criteria for defining multipotent mesenchymal stromal cells. The International Society for Cellular Therapy position statement. Cytotherapy.

[9] Keating A (2012). Mesenchymal stromal cells: new directions. Cell Stem Cell.

[10] Moschidou D (2012). Valproic Acid Confers Functional Pluripotency to Human Amniotic Fluid Stem Cells in a Transgene-free Approach. Mol Ther.

[11] De Coppi P (2007). Isolation of amniotic stem cell lines with potential for therapy. Nature Biotechnology.

[12] Moschidou D (2013). Human mid-trimester amniotic fluid stem cells cultured under embryonic stem cell conditions with valproic acid acquire pluripotent characteristics. Stem Cells Dev..

[13] Langer D, Ikehara Y, Takebayashi H, Hawkes R, Zimmermann H (2007). The ectonucleotidases alkaline phosphatase and nucleoside triphosphate diphosphohydrolase 2 are associated with subsets of progenitor cell populations in the mouse embryonic, postnatal and adult neurogenic zones. Neuroscience.

[14] Kim YH, Yoon DS, Kim HO, Lee JW (2012). Characterization of different subpopulations from bone marrow-derived mesenchymal stromal cells by alkaline phosphatase expression. Stem Cells Dev..

[15] Kenmotsu M, Matsuzaka K, Kokubu E, Azuma T, Inoue T (2010). Analysis of side population cells derived from dental pulp tissue. Int Endod J.

[16] Guerrero-Esteo M, Sanchez-Elsner T, Letamendia A, Bernabeu C (2002). Extracellular and cytoplasmic domains of endoglin interact with the transforming growth factor-beta receptors I and II. J. Biol. Chem..

[17] Wang X, Dai J (2010). Concise review: isoforms of OCT4 contribute to the confusing diversity in stem cell biology. Stem Cells.

[18] Gang EJ, Bosnakovski D, Figueiredo CA, Visser JW, Perlingeiro RCR (2007). SSEA-4 identifies mesenchymal stem cells from bone marrow. Blood.

[19] Schäck LM (2016). Expression of CD24 in Human Bone Marrow-Derived Mesenchymal Stromal Cells Is Regulated by TGFβ3 and Induces a Myofibroblast-Like Genotype. Stem Cells Int.

[20] Takedachi M (2012). CD73-generated adenosine promotes osteoblast differentiation. J. Cell. Physiol..

[21] Ode A (2013). CD73/5′-ecto-nucleotidase acts as a regulatory factor in osteo-/chondrogenic differentiation of mechanically stimulated mesenchymal stromal cells. Eur Cell Mater.

[22] Liu H (2009). A subpopulation of mesenchymal stromal cells with high osteogenic potential. J. Cell. Mol. Med..

[23] Mihaila SM (2014). The osteogenic differentiation of SSEA-4 sub-population of human adipose derived stem cells using silicate nanoplatelets. Biomaterials.

[24] Regateiro FS, Cobbold SP, Waldmann H (2013). CD73 and adenosine generation in the creation of regulatory microenvironments. Clin. Exp. Immunol..

[25] Underwood MA, Gilbert WM, Sherman MP (2005). Amniotic fluid: not just fetal urine anymore. J Perinatol.

[26] Colgan SP, Eltzschig HK, Eckle T, Thompson LF (2006). Physiological roles for ecto-5′-nucleotidase (CD73). Purinergic Signal..

[27] Mahamed DA, Mills JH, Egan CE, Denkers EY, Bynoe MS (2012). CD73-generated adenosine facilitates Toxoplasma gondii differentiation to long-lived tissue cysts in the central nervous system. Proc. Natl. Acad. Sci. USA.

[28] Mediero A, Cronstein BN (2013). Adenosine and bone metabolism. Trends Endocrinol. Metab..

[29] Mediero A, Kara FM, Wilder T, Cronstein BN (2012). Adenosine A(2A) receptor ligation inhibits osteoclast formation. Am. J. Pathol..

[30] Hawkins KE (2017). Human amniocytes are receptive to chemically-induced reprogramming to pluripotency. Mol Ther.

