# Supplementary material for: TGFβ-induced osteogenic potential of human amniotic fluid stem cells via CD73-generated adenosine production
Source: Sci Rep. 2017 Jul 26;7:6601. doi: 10.1038/s41598-017-06780-1 (PMC5529586; doi:10.1038/s41598-017-06780-1)
Supplement: Supplementary file 1 — Supplementary information [file 41598_2017_6780_MOESM1_ESM.pdf]

TGFβ-induced osteogenic potential of human amniotic fluid stem cells via CD73-generated adenosine production.

Kwan-Leong Hau, Anna Maria Ranzoni, Filipa Vlahova, Kate Hawkins, Paolo De Coppi, Anna L David, and Pascale V Guillot

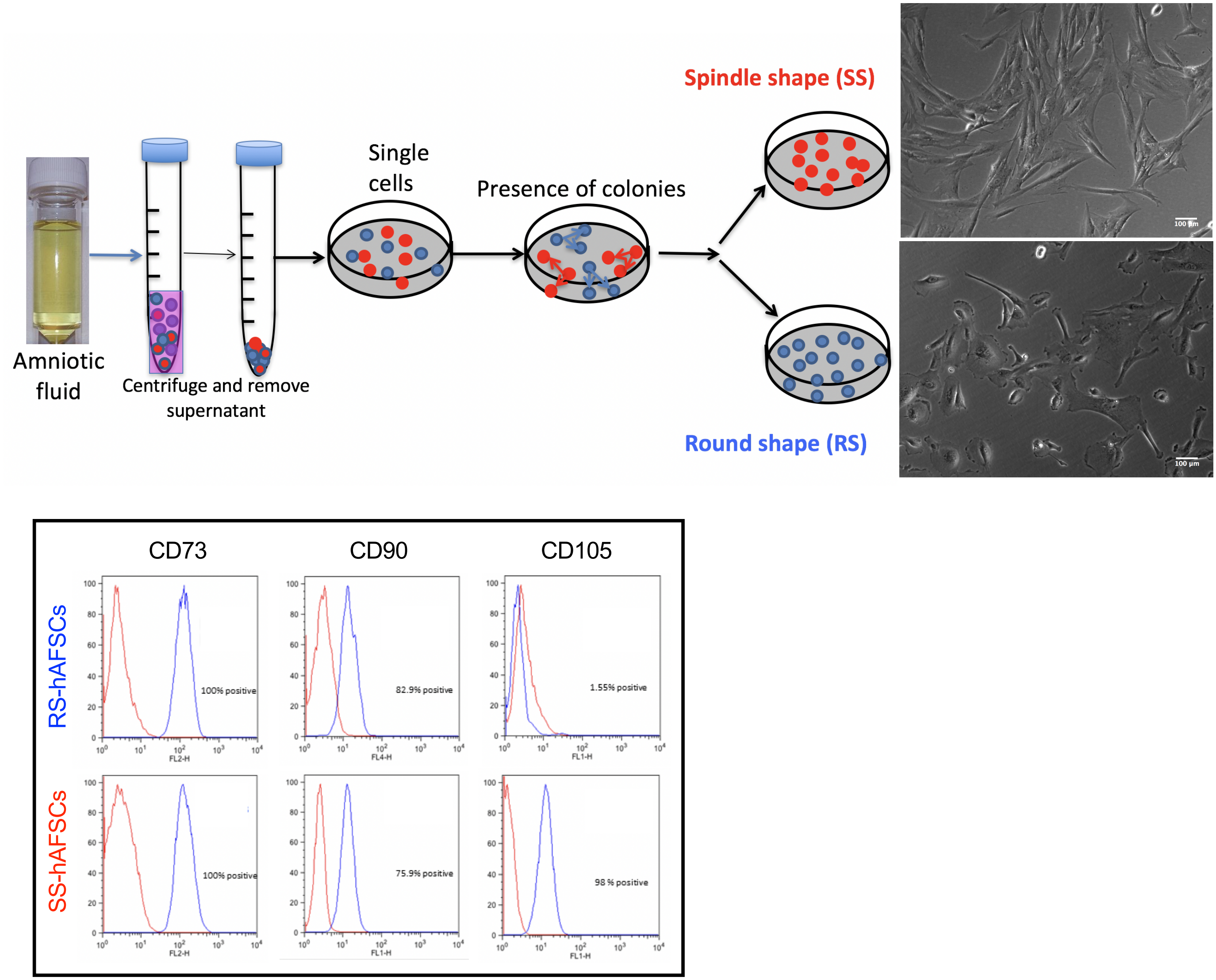

Supplementary Figure 1 | Isolation of RS- and SS-hAFSCs.
Schematic representation of the isolation of RS- and SS-hAFSCs and flow cytometry for the MSC markers CD73, CD90 and CD105 (cells expanded in MT conditions).
